# Early-Age Behaviour of Portland Cement Incorporating Ultrafine Recycled Powder: Insights into Hydration, Setting, and Chemical Shrinkage

**DOI:** 10.3390/ma17225551

**Published:** 2024-11-14

**Authors:** Fei Yang, Yan Ma, Linchang Li, Shuo Liu, Ran Hai, Zheyu Zhu

**Affiliations:** 1 School of Architectural and Civil Engineering, Zhongyuan University of Technology, Zhengzhou 450007, China; 2 Henan Building Materials Research and Design Institute Co., Ltd., Zhengzhou 450052, China; 3 School of Materials Science and Engineering, Yancheng Institute of Technology, Yancheng 224051, China

**Keywords:** Portland cement, hydration kinetics, setting behaviour, chemical shrinkage, ultrafine recycled powder

## Abstract

This study examines the effects of ultrafine recycled powder (URP) obtained from construction and demolition waste on the hydration kinetics, setting behaviour, and chemical shrinkage of Portland cement pastes. The presence of ultrafine particles in the recycled powder provides more sites for nucleation, thereby promoting the hydration process and accelerating the rate of nucleation. As a result, the setting time is reduced while chemical shrinkage is increased. Incorporating URP improves the early-age mechanical properties. When 7.5% URP is added, the highest compressive strength and flexural strength of cement mortar at a curing age of 3 d are 23.0 MPa and 3.7 MPa, respectively. The secondary hydration between the hydration product and reactive silica from URP contributes to gel formation and enhances mechanical property development. This research provides theoretical insights into utilizing recycled powder in cement-based materials and enhances our understanding of its impact on hydration kinetics.

## 1. Introduction

Amongst the various materials employed in human production and daily life [1,2], cementitious material stands out as an extensively utilized building component. Without question, Portland cement holds immense significance as it serves as the predominant type of cementitious material deployed [3]. Nevertheless, with rapid modernization comes an increase in demolitions of aging structures. Consequently, these dismantled edifices now represent valuable solid waste resources. At present, construction solid waste finds initial reuse through recycling into aggregates or powders [4,5,6]. Henceforth, there emerges an urgent need to explore diverse and efficient avenues for utilizing such construction debris.

In recent times, there has been a growing trend of utilizing recycled aggregate derived from construction solid waste in the production of recycled concrete [4,5]. While it is acknowledged that the quality of recycled aggregate may not match that of natural aggregates and they tend to have higher water absorption rates, there are still advantages in using these eco-friendly materials for creating recycled concrete [7,8]. Zhang et al. conducted a study on the impact of heat-treated recycled aggregates on concrete properties [9]. Their findings revealed that heat treatment can effectively reduce the usage of relative mortar bonded to the surface of the waste-recycled aggregate, resulting in a denser microstructure within the concrete and improved mechanical performance. In order to promote sustainable development in building materials, Xu et al. explored the use of recycled powder for manufacturing fibre-reinforced geopolymer material [10]. They discovered that incorporating fibres helped mitigate shrinkage behaviour and reduced cracking in geopolymers, ultimately enhancing its compressive and flexural strengths. This research introduced an innovative approach towards recycling construction solid waste and producing environmentally friendly materials. Leng et al., on their part, employed carbonized waste construction aggregates to prepare ultra-high performance concrete (UHPC) [11]. The results demonstrated that carbonization treatment proved effective in addressing physical and mechanical limitations associated with recycled aggregate while also improving the basic properties, microscopic characteristics, and durability aspects of UHPC. Previous studies have consistently shown how solid wastes originating from construction and demolition activities can be successfully repurposed within cement and concrete applications.

The early characteristics of cement, such as initial hydration, chemical contraction, setting performance, and mechanical strength, hold great significance [12,13,14]. Moreover, the regulation of these properties during the early stages of cement pastes, particularly the hydration process, plays a crucial role in achieving diverse applications for cementitious materials [13,15]. One commonly employed approach involves incorporating different types of chemical additives [16]. Another promising approach is the introduction of ultrafine particles, e.g., nano SiO_2_ and nano TiO_2_, in cement systems [17,18]. In recent times, there has been a significant focus on utilizing solid waste to produce ultrafine powders and incorporating them into cement pastes for the purpose of regulating early hydration [19,20]. He et al. employed wet grinding technology to modify lime mud with sodium hexametaphosphate and utilized it as a mineral additive in cement mortar [21]. They discovered that the optimal amount of sodium hexametaphosphate was found to be 1%. At this level, an effective bonding property between modified lime mud containing ultrafine particles and hydration products can be achieved. Additionally, when 10% modified lime mud was added, the resulting cement mortar exhibited remarkable rheology and mechanical properties. Wang et al. examined the impact of wet-milled calcined gangue on the hydration process and microstructure of phosphogypsum-based cementitious material [22]. They observed that the ultrafine powder derived from calcined gangue demonstrated superior dispersion and dissolution characteristics, leading to accelerated hydration rates and reduced setting time. This improvement facilitated increased availability of active aluminates and silicates for further hydration reactions, consequently enhancing strength development. Henceforth, employing ball milling techniques to prepare ultrafine recycled powder for improving or controlling early cement hydration is highly intriguing.

Here, this paper’s focus revealed the effect of recycled powder containing ultrafine particles on the hydration process, setting behaviour, and composition of reaction products in Portland cement. We assessed the early hydration of cement pastes with varying amounts of URP using tests for hydration heat and chemical shrinkage. To understand the nucleation-growth during hydration, we established a model for hydration kinetics. Additionally, we evaluated the impact of URP on key properties such as setting time and mechanical strength in early-stage cement pastes. The mineralogical and chemical compositions of the hydrated products after 3 d were determined through X-ray diffraction and thermal analysis techniques.

## 2. Experiment Details

### 2.1. Materials

**Figure 1:**
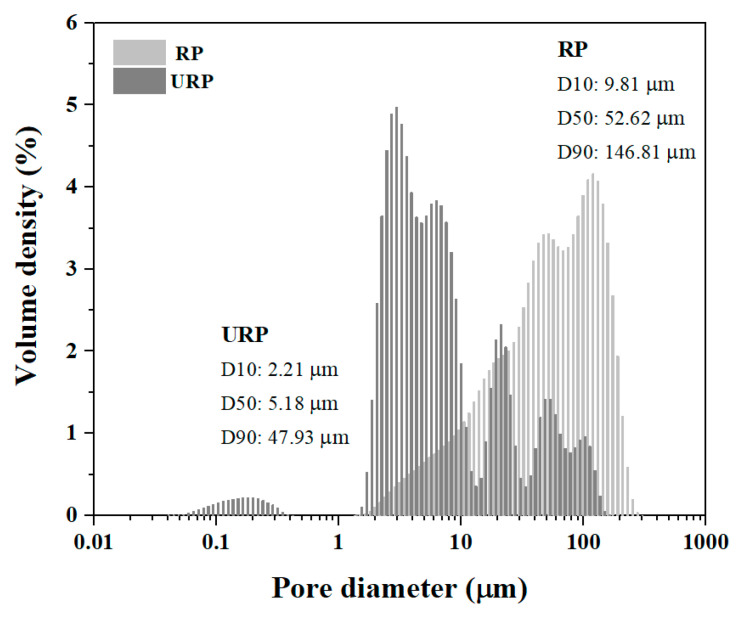
Particle size distribution of RP and URP.

The experimental materials utilized in this study consist of 42.5 Grade ordinary Portland cement (OPC) and ultrafine recycled powder (URP). The OPC was procured from Conch Cement Plant (Zhengzhou, China), while the URP was sourced from a nearby demolition site. Table 1 is the chemical analysis results of OPC and URP by XRF technique. The higher LOI observed in URP can be attributed to its significant CaCO_3_ content, which originates from carbonation behaviour exhibited by hydration products as well as calcite present in the broken aggregate [23,24]. The wet milling method proposed in previous literature [21,25] was employed to obtain the collected recycled powder (RP), with Figure 1 illustrating the particle size distribution outcomes for RP and URP. As they reported, wet milling technology enables effective dispersion of powders, which in turn results in ultra-fine particle sizes. From it, the particle size of URP is significantly smaller than the RP particle size. In detail, the particle parameters, including D_10_, D_50_, and D_90_, of RP are 9.81 μm, 52.62 μm, and 146.81 μm, while those of URP are 2.21 μm, 5.18 μm, and 47.93 μm. In Table 1, the LOI is the volume percentage content of the sample that can maintain the minimum oxygen concentration required for combustion.

### 2.2. Preparation Method of Samples

Table 2 presents the composition of the cement mortar mixture used in this study. The URP content ranged from 0% to 10%. The different URP dosages resulted in four types of cement mortars, namely P-U0, P-U5, P-U7.5, and P-U10. For the preparation of these cement mortars, a water to binder ratio (W/B) of 1:2 and a binder to sand ratio (B/S) of 1:3 was selected. To evaluate the early-age mechanical properties, cement mortar specimens measuring 40 mm × 40 mm × 160 mm were utilized. Additionally, microscopic characteristics were examined by preparing cement pastes for analysis purposes.

### 2.3. Testing Methods

#### 2.3.1. Chemical Shrinkage

Based on the ASTM C1608-17 standard and relevant literature [26,27], we conducted a chemical shrinkage measurement with varying dosages of URP at the early stage. The theoretical basis of this experiment is the absolute volume difference caused by the volume imbalance between reactants and products, which is measured by the change in liquid level. The experiment was performed in a water bath box at a temperature of 23 °C, and for each group, we ensured accuracy by using at least three replicate samples to obtain the mean value.

#### 2.3.2. Setting Time and Compressive Strength

In order to comply with the Chinese standard GB/T 1346-2011, we utilized the Vicat apparatus to measure the impact of URP addition on the setting time of fresh cement paste. Additionally, following the guidelines outlined in the Chinese standard GB/T 17671-1999, we employed a fully automated machine tester (WHY-200, Hualong, Shanghai, China) to determine strengths of hardened cement mortar. Six replicate samples were analysed for each group and an average value was calculated.

#### 2.3.3. Hydration Heat Test

The exothermic hydration of cement pastes with different URP contents was examined in-situ by isothermal calorimetry. According to the ASTM C1702-17 standard, an eight-channel TAM Air isothermal calorimeter (TAM 83 Air, Thermometric, New Castle, DE, USA) was used to record the heat evolution during the hydration process.

#### 2.3.4. Hydration Kinetics

In order to gain a better understanding of the rate at which crystals dissolve and react, researchers have developed and refined Avrami’s theory [28,29,30]. Building upon this theory, we have introduced the Johnson-Mehl-Avrami-Kolmogorov (JMAK) model to assess how replacing PC with URP affects the hydration process of cement paste. By utilizing Equation (1) below, we can calculate the parameter for degree of hydration (α).

(1) $$\alpha =\frac{Q_{i}}{Q_{t}}$$

where, *Q_i_* (J/g) is the heat release at the real-time point, and *Q_t_* (J/g) is the total heat release within the studied time range.

As crucial factors in assessing the kinetics of hydration, the Avrami exponent (N) and growth rate (K) can be determined using Equation (2), which is derived from the JMAK model:

(2) ln[−ln(1−α)]=Nlnt+lnK

Herein, α is the hydration degree and *t* is the hydration time (min). It is important to mention that Equation (2) can be transformed into the form of a linear function, namely y = Ax + B. Herein, ln⁡[−ln⁡(1−α)] is on the x-axis and lnt is on the y-axis. By representing it as a linear graph, one can determine the values of N and K. In this linear function, the slope (A) represents the Avrami exponent while the intercept (B) corresponds to the logarithm of the growth rate (lnK, s^−1^).

#### 2.3.5. X-Ray Diffraction Analysis

The mineral composition of the 3-d cured cement sample was analysed by using an X’Pert3 Powder X-ray diffractometer with Cu Kα radiation (PANalytical, Holland, The Netherlands). At the 40 kV working voltage and 30 mA working current, the whole testing procedure was performed from 10° to 60° 2θ at a scanning rate of 3.6°/min.

#### 2.3.6. Thermogravimetric Analysis

With the use of NETZSCH-STA 449C thermal analyser (NETZSCH, Selb, Germany), the chemical composition of the 3-day cured cement sample was characterized. Under the protection of a nitrogen atmosphere, the temperature ranges from 30 °C to 1000 °C at the heating rate of 10 °C/min.

## 3. Results and Discussion

### 3.1. Heat Evolution

**Figure 2:**
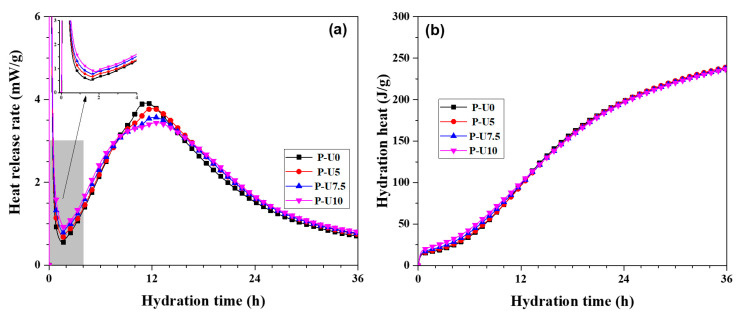
Heat release rate and hydration heat of cement pastes. (**a**) Heat release rate; (**b**) hydration heat of cement pastes.

Figure 2 shows the effect of URP dosage on the heat release rate and hydration heat. The hydration process of silicate cement mainly includes induction, acceleration, and deceleration periods [12,31]. From it, all cement pastes exhibit similar exothermic rate characteristics, implying that the URP did not fundamentally change the hydration process. Notably, the usage of URP significantly shortened the hydration induction period, facilitating the advancement of the acceleration period. This suggests that the ultrafine particles of a size from 0.05 μm to 0.4 μm in URP promoted the cement hydration, which was consistent with the findings of Kontoleontos et al. [32] and Li et al. [33]. Moreover, the exothermic peak of hydration between the acceleration and deceleration periods decreased slightly with increasing URP dosage, which was the reduction of clinker in the system [34]. Figure 2b presents the cumulative hydration heat of cement pastes within the first 36 h. As shown in it, the cumulative hydration heat of cement pastes with different dosages of URP is comparable. In detail, the hydration heat of P-U0, P-U5, P-U7.5, and P-U10 samples is 237.80 J/g, 239.15 J/g, 237.90 J/g, and 236.94 J/g, respectively. The close hydration heat between different cement pastes indicates that the secondary hydration between the SiO_2_ in the URP and the Ca(OH)_2_ (CH) generated from hydration might happen. Overall, the control of the cement hydration process, especially the acceleration period, can be achieved through the introduction of URP. To achieve a deeper understanding about the role of URP on the hydration of cement paste, it is essential to establish the hydration kinetics model.

### 3.2. Hydration Kinetics

**Figure 3:**
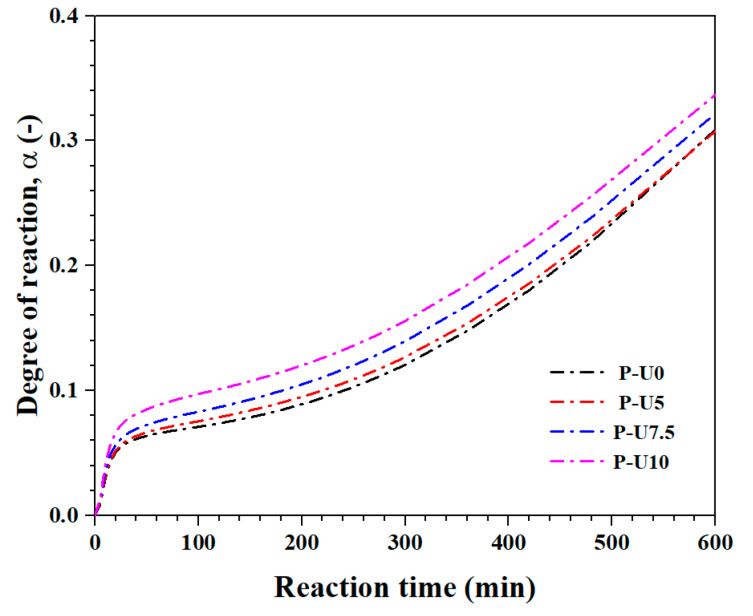
Hydration degree of cement pastes with different dosages of URP.

The hydration progress of cement pastes with varying amounts of URP is illustrated in Figure 3. It can be observed that the hydration progress of all cement pastes increased gradually over time, indicating the advancement of the hydration reaction [35,36]. The inclusion of URP resulted in an increase in early-age hydration progress, which was directly proportional to the amount of URP present. Specifically, for samples P-U0, P-U5, P-U7.5, and P-U10, the corresponding hydration progresses were measured at 0.31, 0.31, 0.32, and 0.34, respectively. This increase in hydration progress can be attributed to the ultrafine size of URP particles, which act as nucleation sites and accelerate the process of hydration [37,38]. In order to further investigate how URP affects the kinetics of cement paste hydration, kinetic models were developed to determine both Avrami exponent (N) and growth rate (K).

**Figure 4:**
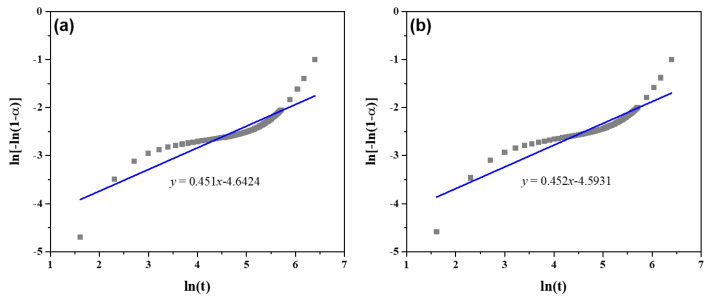

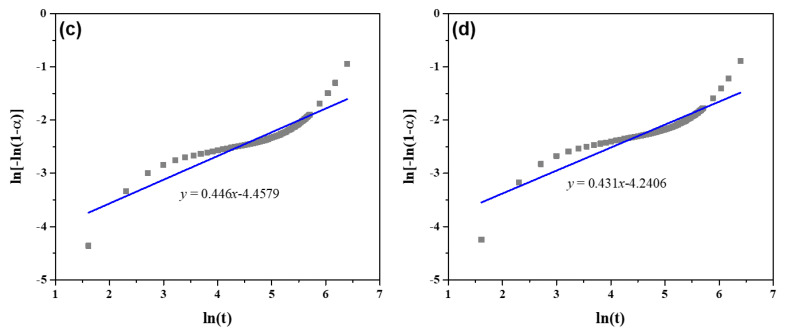
Avrami’s plot of cement pastes with different dosages of URP. (**a**) P-U0; (**b**) P-U5; (**c**) P-U7.5; (**d**) P-U10.

Figure 4 illustrates the Avrami’s plot of cement pastes containing varying amounts of URP. It can be observed that all the cement pastes exhibit similar Avrami characteristics. The key parameters of reaction kinetics were determined by fitting the Avrami’s plot linearly. Table 3 presents the impact of URP on the N and K values of cement pastes after a 10-h curing period. The findings indicate that the N values range from 0.431 to 0.452, which falls within the range reported in previous studies [39,40]. This suggests that the reaction type of cement paste remains unchanged with the incorporation of URP. Additionally, it is evident that an increase in URP content leads to a higher value for K. This can be attributed to the presence of ultrafine particles in URP, which provide more nucleation sites and subsequently reduce nucleation barriers while accelerating nucleation rates. Consequently, this improvement in hydration process contributes to enhanced setting and mechanical properties development. It is noteworthy to point that an increase in URP microparticles could be detrimental as saturation of these particles could occur when the hydration rate decreases.

### 3.3. Chemical Shrinkage

**Figure 5:**
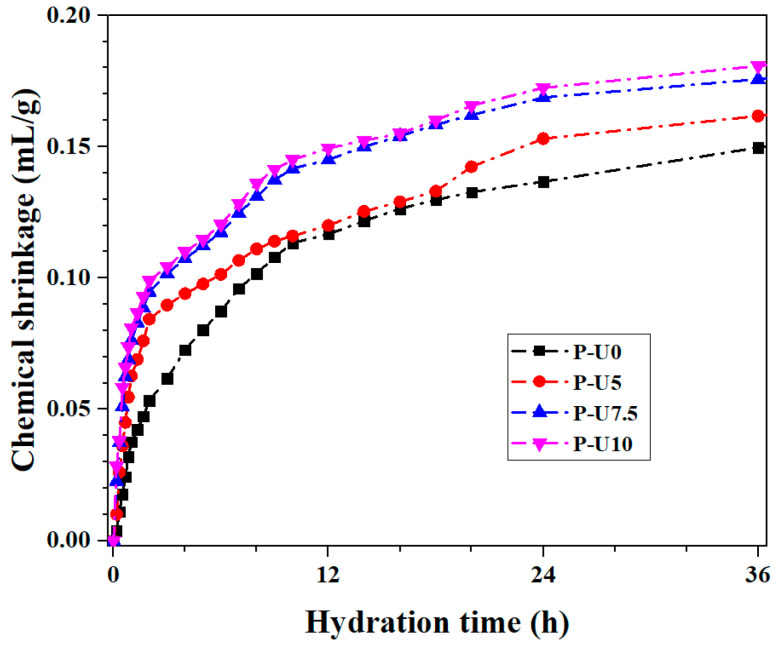
Chemical shrinkage of cement pastes with different dosages of URP.

Figure 5 illustrates the outcomes of early-age chemical shrinkage in cement paste containing various amounts of URP. Previous studies [14,41] have indicated that the chemical shrinkage of cement-based materials can serve as an indicator for their hydration process and degree of hydration to some extent. From Figure 5, it can be observed that the chemical shrinkage gradually increases with higher URP content. The incorporation of a small amount of URP can have a positive effect and accelerate cement hydration. However, excessive URP dosage sacrifices cement dosage, which would be not conducive to the development of comprehensive performance. Within the first 4 hours, there is a noticeable rise in chemical shrinkage, which aligns well with the cumulative hydration heat findings discussed in Section 3.1. After 36 h of curing, the chemical shrinkage values for P-U0, P-U5, P-U7.5, and P-U10 samples are recorded as 0.1497 mL/g, 0.1616 mL/g, 0.1756 mL/g, and 0.1807 mL/g, respectively. This sustained elevation in chemical shrinkage suggests that ultrafine particle-sized URP has a significant influence on the hydration process of cement paste.

### 3.4. Setting Time

**Figure 6:**
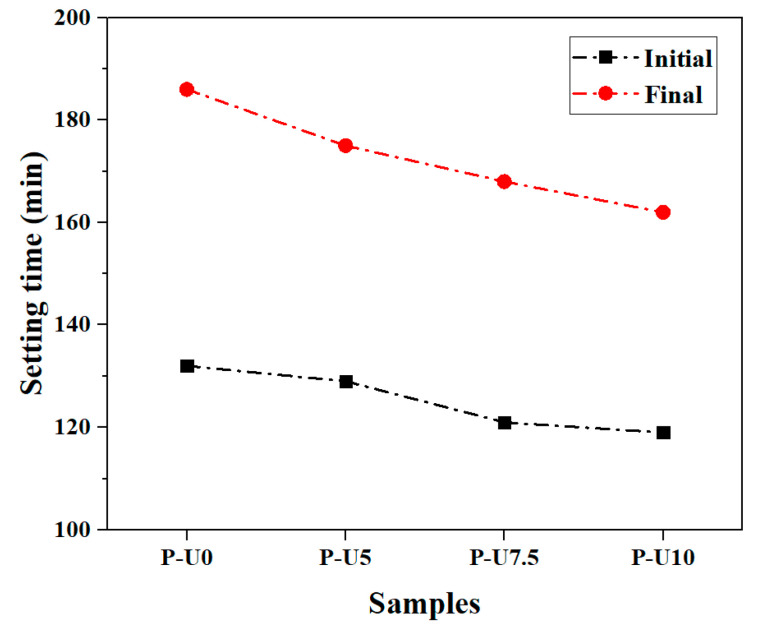
Setting time of cement pastes with different dosages of URP.

**Figure 7:**
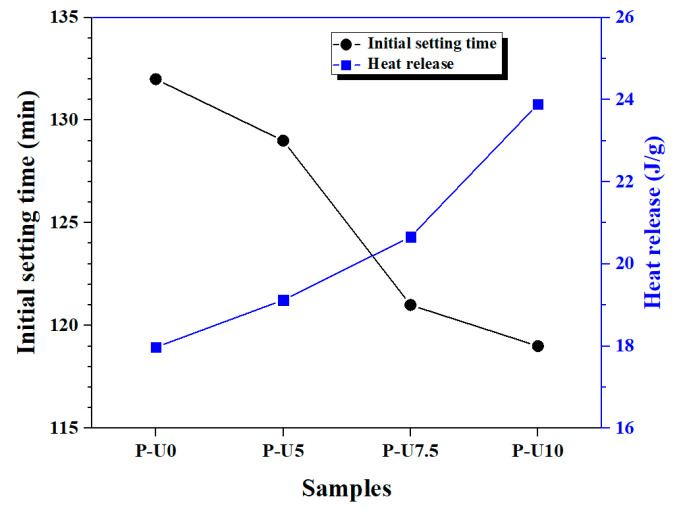
Variation of heat released during the initial setting period.

Figure 6 illustrates the initial and final setting time of cement pastes with varying amounts of URP. It can be observed that the P-U0 paste, which does not contain any URP, had an initial setting time of 132 min and a final setting time of 186 min. The addition of URP gradually reduced both the initial and final setting times. When the dosage of URP reached 10%, the P-U10 paste exhibited an initial setting time of 119 min and a final setting time of 162 min. This reduction in setting time confirms that URP enhances the hydration process in cement paste. To gain further insights into the relationship between heat release and setting time, Figure 7 presents a comparison between the initial setting time and cumulative hydration heat during this period. It is evident that there exists an inverse correlation between the initial setting time and cumulative heat release for cement paste. This inverse relationship suggests that faster reaction processes result in higher heat release rates and shorter overall setting times. In summary, by incorporating URP, it becomes possible to regulate both reaction processes and setting behaviour in cement paste effectively.

### 3.5. Compressive and Flexural Strengths

**Figure 8:**
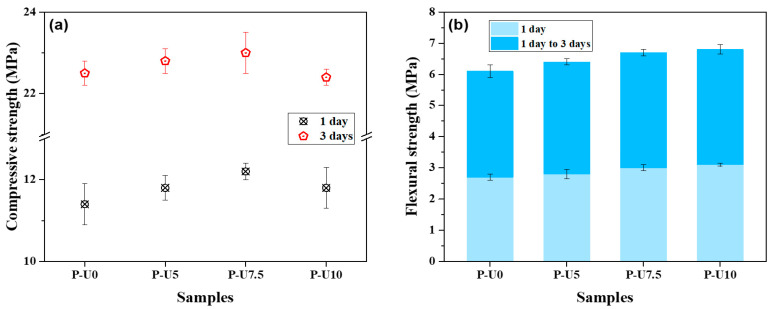
Effect of URP on the compressive strength and flexural strength of cement mortars. (**a**) Compressive strength; (**b**) flexural strength of cement mortars.

Figure 8 illustrates the compressive and flexural strengths of hardened cement mortars containing varying amounts of URP. As shown in Figure 8a, the addition of URP slightly increased the compressive strength of the cement mortar. For example, P-U0 sample exhibited compressive strengths of 11.4 MPa at 1 day and 22.5 MPa at 3 d curing ages. The highest compressive strengths were achieved by P-U7.5 sample with an inclusion of 7.5% URP. At this usage level, the promotion of URP to the hydration was optimum and the sacrificial gel generation was relatively slight. Consequentially, P-U7.5 sample exhibited good comprehensive mechanical properties. When the dosage was increased to 10%, P-U10 samples showed comparable compressive strength to P-U0 samples. The development of flexural strength followed a similar trend as that of compressive strength, with one notable difference being that there was no significant decrease in flexural strengths for P-U10 samples. The mechanical properties enhancement can be attributed to URP’s promotion of the early-age reaction process, which also facilitated the formation of reaction products and densification of microstructures [42].

### 3.6. Hydrate Composition Analysis

**Figure 9:**
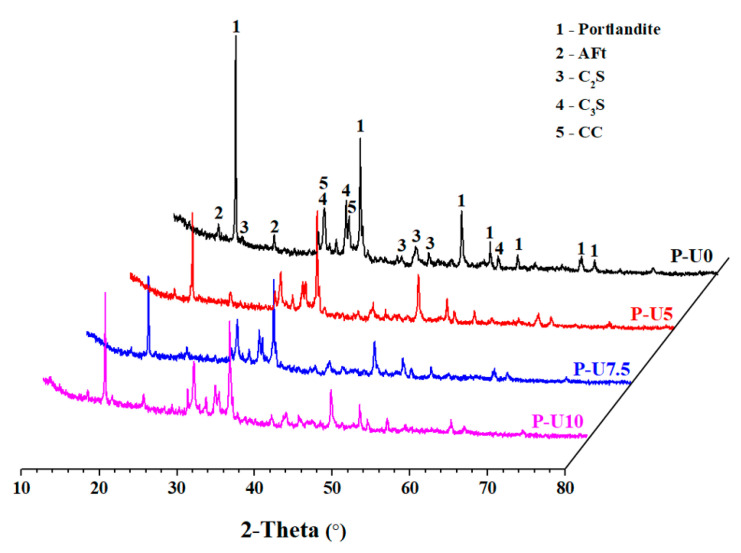
XRD patterns of 3-day cured cement pastes with different dosages of URP.

Figure 9 illustrates the impact of URP on the mineral composition of cement pastes cured for 3 days. The results show that common hydration products, such as calcium hydroxide (CH) and ettringite (AFt), were present in all cement pastes. Additionally, there were unhydrated mineral phases, namely C_2_S and C_3_S, observed in the cement paste. The presence of calcite (CC) can be attributed to potential carbonation behaviour of CH. These findings align with previous studies on silicate cement hydration products [43,44], indicating that the introduction of URP did not significantly alter the reaction products formed during cement paste formation. Notably, an increase in URP content led to a gradual decrease in diffraction peak intensity at 18 °C, 35 °C, and 47 °C 2θ angles for CH. This suggests changes occurring within CH when URP dosage is increased. It has been reported [19] that ultrafine recycled powder contains reactive silica, which undergoes secondary hydration with CH and contributes to mechanical property development. Another factor contributing to reduced CH generation was the decreased use of Portland cement during paste preparation.

**Figure 10:**
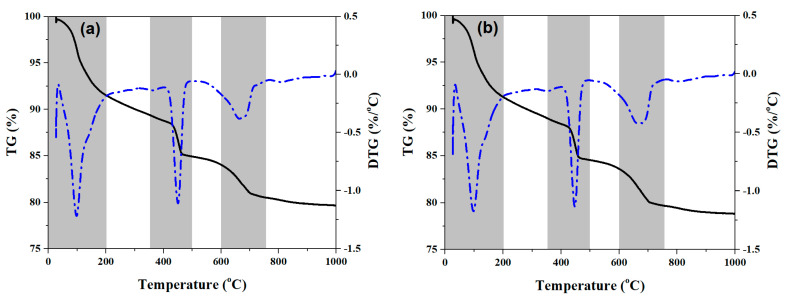

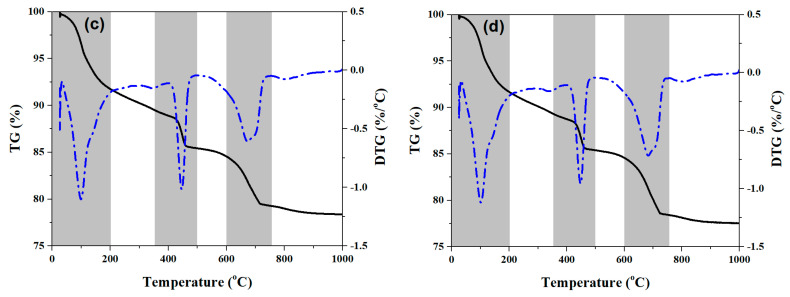
TGA data of 3-day cured cement pastes. (**a**) P-U0; (**b**) P-U5; (**c**) P-U7.5; (**d**) P-U10.

Figure 10 illustrates the TG and DTG curves of cement pastes cured for 3 d, containing different amounts of URP. Upon comparing these thermal analysis findings, it becomes evident that all the cement pastes exhibit similar characteristics in terms of TG and DTG curves, suggesting a potential similarity in composition of hydration products. In Figure 10, the peaks observed within the temperature range of 200 °C are attributed to physically bound water and free water present in both C-S-H gel and AFt phases [45,46]. The mass loss occurring between 350 °C and 500 °C can be attributed to the dehydroxylation behaviour of portlandite, which is closely associated with CH content generated during cement paste formation [47]. Additionally, due to possible carbonation effects [38,48,49,50], calcite formation leads to distinctive peaks observed between temperatures ranging from 600 °C to 750 °C. In addition, calcite formation at such an early stage may be more related to potential inadequate sample storage than to actual hydration processes. To quantify variations within these three temperature ranges accurately, mass loss rates (wt.%) were calculated based on TG curve data as presented in Table 4.

It is intriguing to note that the initial stage exhibited an increase followed by a decrease in mass loss as the URP content varied, suggesting that the inclusion of URP contributed to the production of hydration products. Simultaneously, there was a gradual reduction in mass loss within the temperature range of 350 °C–500 °C with increasing URP dosage. This aligns with the observed alteration in intensity of diffraction peaks for CH in XRD pattern. In theory, CH has a negative effect on strength, indicating it does not affect the mechanical properties of the cement and at the same time reduces production costs. The mass loss between 600 °C and 750 °C noticeably escalated alongside higher URP content. This implies that incorporating URP led to greater gel formation and an increase in calcite quantity. Overall, integrating URP into cement paste facilitated hydration product formation, consequently influencing macroscopic property development. In addition, the potential application of research results is important for the nuclear industry, as there are large-scale retirement projects there, and the methods used and results obtained may have sensitive impacts.

## 4. Conclusions

Employing ball milling techniques to prepare ultrafine recycled powder for improving or controlling early cement hydration is highly intriguing. This study investigated the role of ultrafine recycled powder (URP) on the hydration kinetics, setting behaviour and chemical shrinkage of cement pastes. Based on the above mentioned information, the conclusions can be obtained as the following:

(1) The incorporation of URP promoted the hydration of cement paste, resulting in an earlier acceleration period. It was believed from the hydration kinetics model that the addition of URP did not change the reaction type of the cement paste, but it provided more nucleation sites, contributing to the acceleration of nucleation rate.
(2) Due to the promotion of URP to the hydration process of cement paste, the setting time of fresh cement paste was shortened and the early-age chemical shrinkage was increased. At the same time, the early-age mechanical properties first increased and then slightly decreased with the content of URP. The optimum usage of URP was 7.5%, the 3-day compressive and flexural strengths were 23.0 MPa and 3.7 MPa at this level.
(3) Combined with the results of mineral and chemical compositions, secondary hydration occurred between the hydration product (calcium hydroxide) and the reactive silica from URP. This is beneficial for the formation of gel and the development of mechanical properties. In total, the URP can be used for the controlling in the hydration process of cement. Integrating URP into cement paste facilitated hydration product formation, consequently influencing macroscopic property development.

## Figures and Tables

**Table 1:** Chemical components of the OPC and URP (wt.%).

Oxide	CaO	SiO_2_	Al_2_O_3_	Fe_2_O_3_	TiO_2_	MgO	K_2_O	Na_2_O	LOI
OPC	64.6	20.3	5.1	3.7	0.2	0.9	0.5	0.1	1.5
URP	39.8	14.3	5.9	2.4	0.1	5.2	0.3	0.2	30.2

**Table 2:** Mixture composition of cement mortars (wt.%).

Sample	OPC (g)	URP (g)	W/B	B/S
P-U0	100	0	1:2	1:3
P-U5	95	5	1:2	1:3
P-U7.5	92.5	7.5	1:2	1:3
P-U10	90	10	1:2	1:3

**Table 3:** Avrami’s parameters of cement pastes with different dosages of URP.

Samples	P-U0	P-U5	P-U7.5	P-U10
N	0.451	0.452	0.446	0.431
K	6.634 × 10^−3^	10.121 × 10^−3^	11.587 × 10^−3^	14.399 × 10^−3^

**Table 4:** Mass loss (wt.%) of cement pastes in 200 °C, 350–500 °C, and 600–750 °C.

Sample	200 °C	350–500 °C	600–750 °C
P-U0	8.49	4.5	3.49
P-U5	8.72	4.51	3.88
P-U7.5	8.55	4.12	5.26
P-U10	8.32	3.97	6.13

## Data Availability

The original contributions presented in the study are included in the article, further inquiries can be directed to the corresponding authors.
